# Hidden Nematode Causing Recurrent Pancreatitis: A Case Report

**DOI:** 10.7759/cureus.11835

**Published:** 2020-12-01

**Authors:** Khalil Zahida, Prakash Joseph, Girisha Balaraju, Saad Rashid Mohammad Al Kaabi, Vamanjore A Naushad

**Affiliations:** 1 General Internal Medicine, Hamad Medical Corporation, Doha, QAT; 2 General Internal Medicine, Hamad General Hospital/Hamad Medical Corporation, Doha, QAT; 3 Gastroenterology, Hamad General Hospital/Hamad Medical Corporation, Doha, QAT

**Keywords:** pancreatitis, hepatobiliary, ascariasis

## Abstract

Even though hepatobiliary ascariasis has been found to cause pancreatitis, it is rare in Qatar and other countries in the Middle East. In this report, we present a case of biliary duct ascariasis causing recurrent pancreatitis. A 46-year-old woman from the Philippines presented with recurrent clinical and biochemical features of acute pancreatitis and was found to have hepatobiliary ascariasis. She was successfully treated with endoscopic retrograde cholangiopancreatography (ERCP) and antihelminthic medication. Although hepatobiliary ascariasis as a cause of pancreatitis is rare, it should be considered in patients with recurrent pancreatitis without an obvious cause, especially in those from endemic areas or those who have visited endemic areas.

## Introduction

Hepatobiliary and pancreatic ascariasis (HPA) was first described as a clinical entity from Kashmir, India in 1985. HPA is caused by the invasion and migration of the nematode *Ascaris lumbricoides* into the biliary tract and pancreatic duct. Its clinical presentations include biliary colic, cholangitis, cholecystitis, acute pancreatitis, and, rarely, hepatic abscess [1], Bile peritonitis has also been reported [2]. A study from Kashmir, where ascariasis infection is endemic, has reported that in 23% of patients with acute pancreatitis, HPA was found to be the etiological factor [3]. In this report, we discuss a case of recurrent pancreatitis caused by biliary ascariasis.

## Case presentation

A 46-year-old Filipino woman was admitted with acute abdominal pain and vomiting for one day. Her past medical history included being treated for latent tuberculosis with rifampicin and pyridoxine for one month. There were no other comorbidities. Laboratory analysis showed leukocytosis of 13.5 x 10^3^/uL (reference range: 4.0-10 x 10^3^/uL), eosinophil count of 0.7 x 10^3^/uL (reference range: 0.0-0.5 x 10^3^/uL), elevated alanine aminotransferase (ALT) of 126 U/L (reference range: 0-33 U/L), aspartate aminotransferase (AST) of 757 U/L (reference range: 0-32 U/L), bilirubin of 23 umol/L (reference range: 0-21 umol/L), and lipase of >6,000 IU/L (reference range: 13-60 IU/L). Alkaline phosphatase (ALP) was within the normal reference range (Table 1).

**Table 1:** Laboratory results of the patient WBC: white blood cells; ALT: alanine aminotransferase; AST: aspartate aminotransferase

Tests	Normal range	First visit	Second visit	Third visit	Fourth visit
WBC count	4.00-10.00 x 10^3^/uL	13.5	10.0	14.3	16.8
Eosinophil count	0.0-0.5 × 10^3^/uL	0.7	0.7	0.1	0.5
Amylase	13-53 IU/L				1,386
Lipase	8-78 IU/L	>6,000	>800	>800	5,902
Total bilirubin	3.4-20.5 umol/L	23	8.0	26	14
ALT	0-33 U/L	126	16	348	98
AST	0-32 U/L	757	28	883	246
Alkaline phosphatase	35-104 U/L	62	44	71	88

Her transabdominal ultrasound was reported normal. She was diagnosed with acute pancreatitis and was given symptomatic treatment for pain along with intravenous fluids. She was discharged home in two days as her symptoms subsided. Her elevated liver function tests were attributed to antituberculous therapy.

She had another attack of mild pancreatitis two months later; this time, 4 mm calculus was seen at the neck of the gallbladder and a normal common bile duct (CBD). She underwent laparoscopic cholecystectomy during the same admission after the clinical resolution of pancreatitis.

She was readmitted two months after surgery with upper abdominal pain and vomiting. Laboratory workup showed leukocytosis of 14.3 x 10^3^/uL, ALT of 348 U/L, AST of 883 U/L, normal alkaline phosphatase, and lipase of >800 IU/L. Her ultrasound revealed a swollen pancreas with peripancreatic fluid in the right subhepatic space extending to the perinephric area and fatty infiltration of the liver.

Due to recurrent acute pancreatitis, a suspicion of retained gallstone in the biliary tree was raised, and the patient underwent magnetic retrograde cholangiopancreatography (MRCP) with contrast, which showed diffuse thickening of the pancreas with minimal peripancreatic free fluid, inflammatory fat stranding, and secondary thickening of the duodenum, suggestive of acute pancreatitis. A small pocket of fluid collection in the peripancreatic region was also noted. However, no choledocholithiasis was seen. She was discharged home after the improvement in her symptoms.

**Figure 1:**
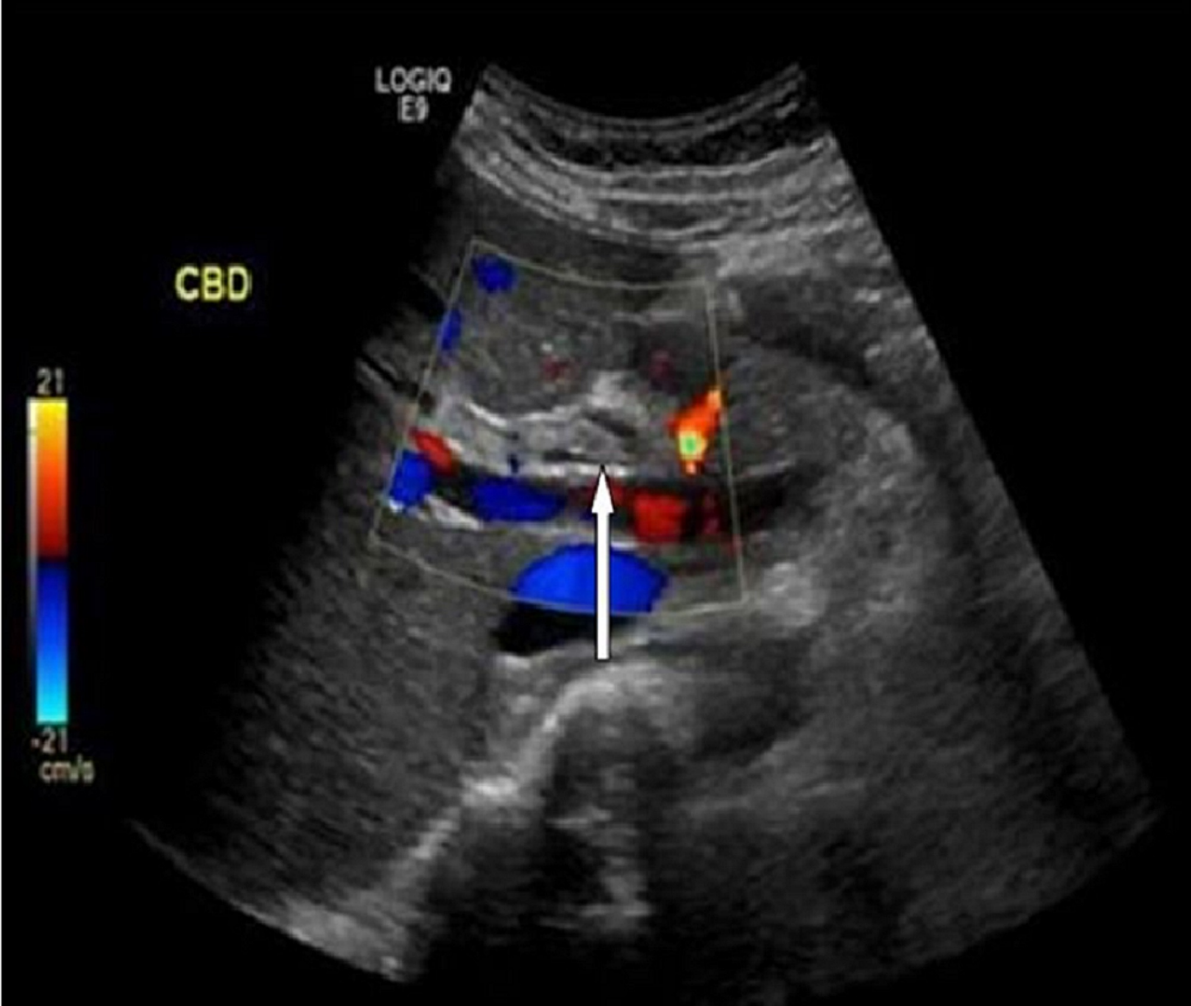
Biliary ultrasound depicting the radiological four-line sign (arrow)

She had a fourth attack of acute pancreatitis after two and a half months of the previous one, without any clinical features of cholangitis. Abdominal ultrasound was repeated, which showed a linear echogenic structure within the CBD. CBD measured 8 mm and intrahepatic biliary radicle dilatation was also noted (Figure 1).

**Figure 2:**
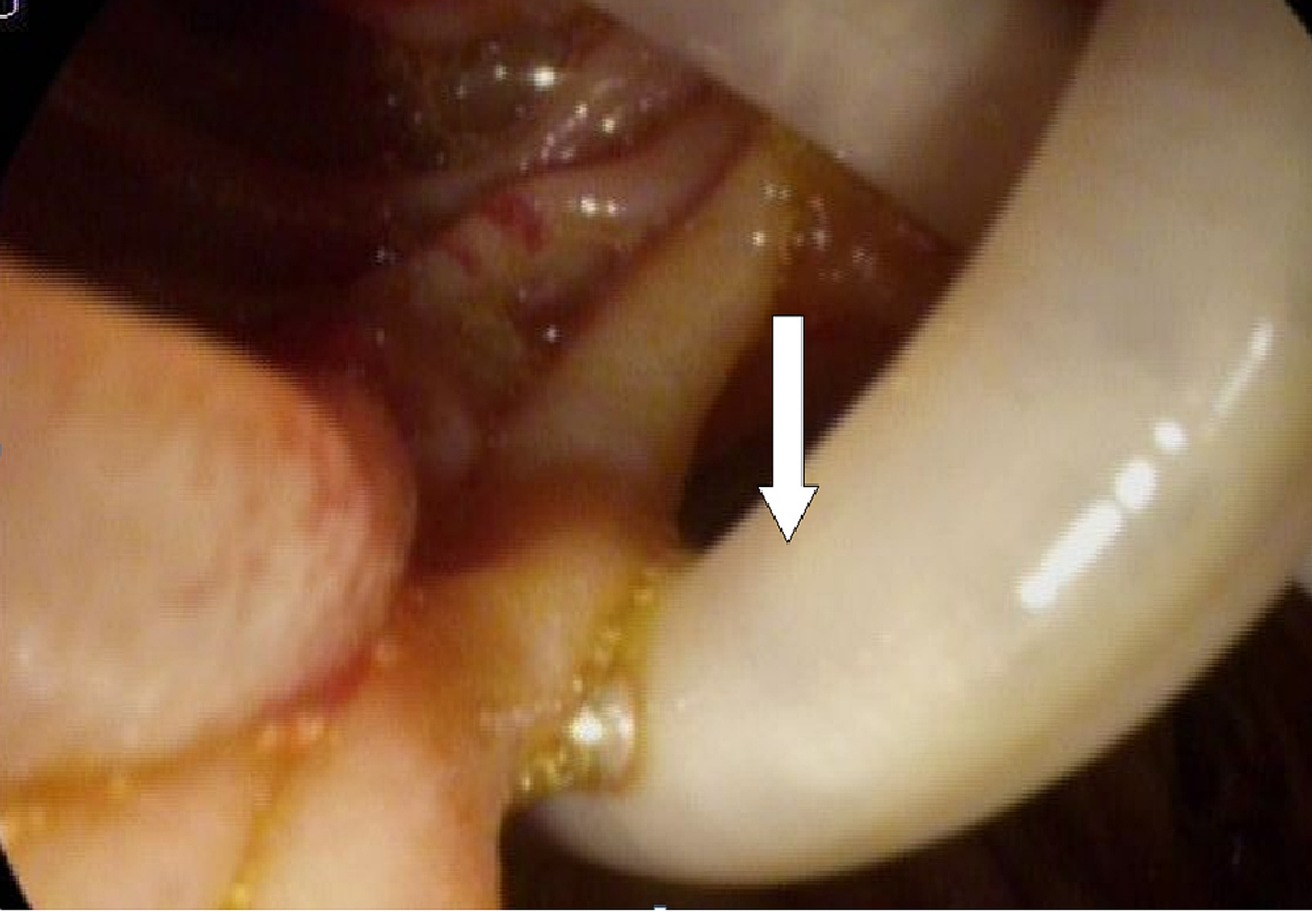
Endoscopic image showing Ascaris worm (arrow) - image 1

**Figure 3:**
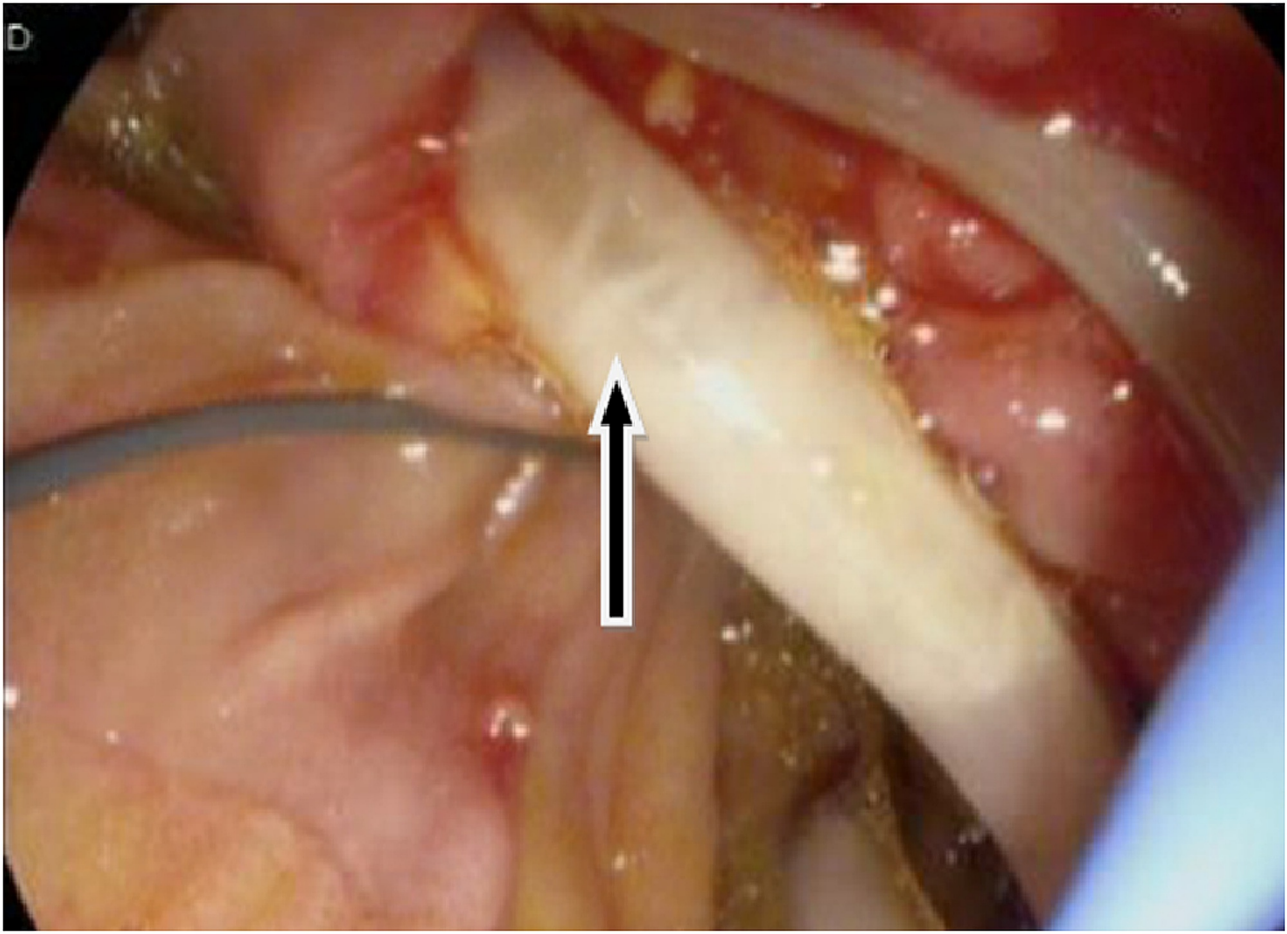
Endoscopic image showing Ascaris worm (arrow) - image 2

**Figure 4:**
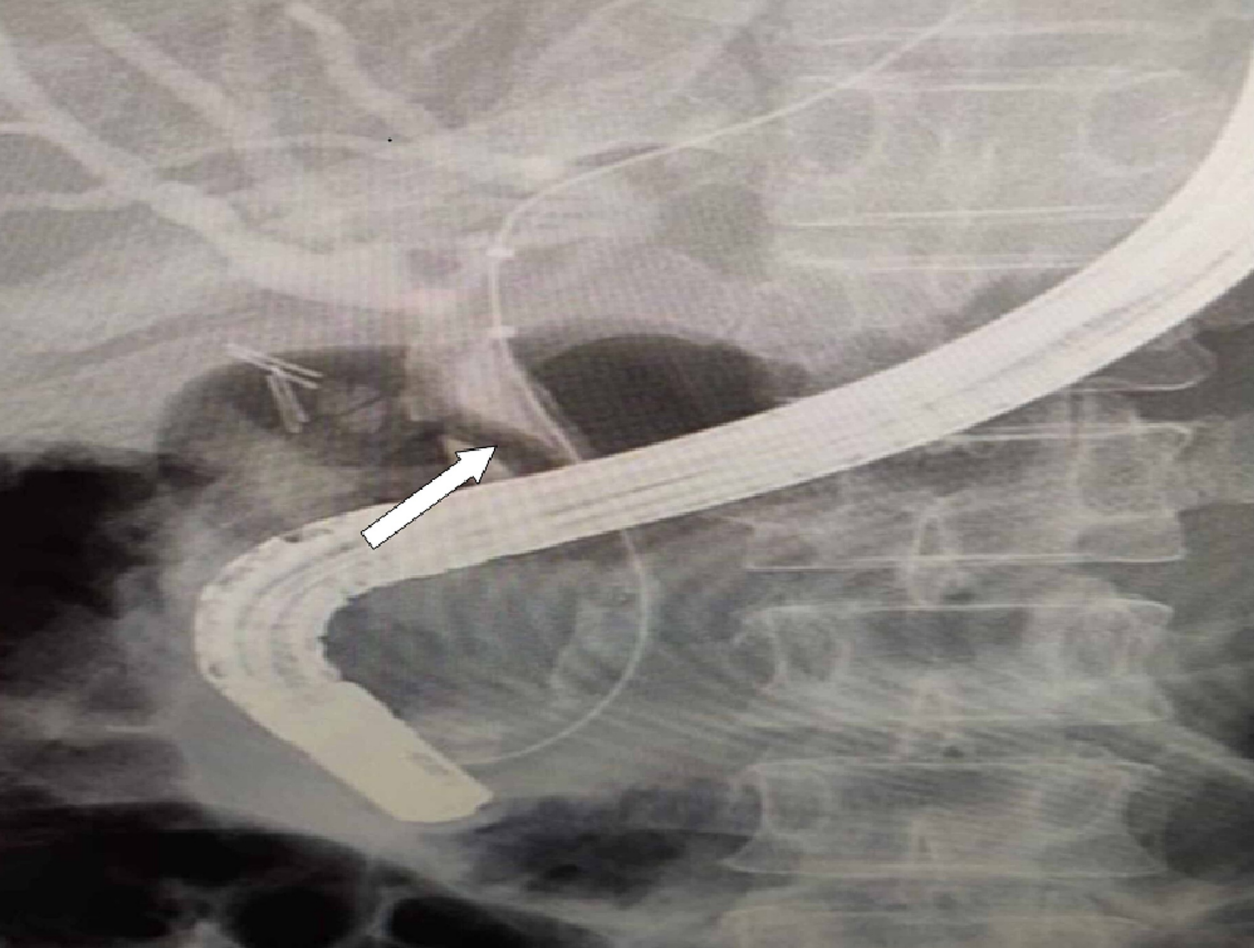
Endoscopic retrograde cholangiopancreatography showing a filling defect in the common bile duct (arrow)

Endoscopic ultrasound revealed two linear echogenic structures within the wall of CBD. Endoscopic retrograde cholangiopancreatography (ERCP) done on the same day showed two long *Ascaris lumbricoides* adult worms in the bile duct (Figures 2, 3, 4).

Biliary sphincterotomy and CBD clearance were done. The patient was treated with a stat dose of albendazole, which had to be repeated after four weeks, and antibiotics for four days. The patient was discharged home on day five, and she was asymptomatic at the time of discharge. She was followed up in the outpatient clinic and was found to be doing fine without any symptoms.

## Discussion

*Ascaris lumbricoides* is one of the most common intestinal parasites worldwide, which is more commonly found in tropical countries where sanitation is poor. Sporadic cases of pancreatic ascariasis have been reported from non-endemic countries like Italy [4], Spain [5], Switzerland [6], and Lithuania [7].

Our patient had recurrent presentations with symptoms and biochemical features of acute pancreatitis. Although a cause was found and sorted out at each presentation, it was only during her fourth visit that *Ascaris lumbricoides* was established as the cause of her pancreatitis.

Studies have shown that recurrent pancreatitis in patients can be attributed to various unusual causes. Sandouk et al. reviewed 300 patients with pancreatic ascariasis in Syria and showed that ultrasonography, together with clinical findings, is the mainstay of diagnosing pancreatic ascariasis [8]. This finding has been echoed by another review of 14 patients with biliary ascariasis [9]. The failure to diagnose ascariasis in our case may have been due to the migration of the worm out of the biliary ducts during the investigations.

Eosinophilia can serve as one of the many diagnostic clues to look for the presence of a helminthic infestation. A retrospective study for the presence of eosinophilia was done on 621 cases positive for parasitic infestation. Out of the 621 cases, *Trichuris trichiura*, *Ascaris lumbricoides*, *Strongyloides stercoralis*, filarial worm, and hookworm accounted for eosinophilia in 88.9%, 87.5%, 63.6%, 53.8%, and 35.5% of the cases respectively [10]. Retrospectively, we found that our patient's eosinophil count was on the higher side on two visits.

The treatment for HPA includes appropriate management of clinical syndromes along with effective antihelminthic medications: albendazole or mebendazole. Endotherapy in HPA is indicated if patients continue to have symptoms on medical therapy or when worms do not move out of ductal lumen by three weeks or die within the ducts [3]. The extraction of worms by ERCP is easier if it is protruding out of the papilla. The worms should be extracted completely because remnants can lead to stone formation. Removal of the worm is usually associated with rapid relief of clinical symptoms [8,11]. Patients should be treated with antihelminthic therapy to eradicate the remaining worms.

## Conclusions

A differential diagnosis of biliary ascariasis as a cause should be considered when a patient presents with recurrent pancreatitis with deranged liver enzymes without any specific cause along with eosinophilia. Endoscopy and endoscopic ultrasound should be considered when no specific cause is found in patients with recurrent pancreatitis. We believe that there is no harm in treating such patients, especially those from endemic areas, with anthelminthic therapy, particularly those with raised eosinophil count on presentation.
